# Implementation Outcomes Assessment of a Digital Clinical Support Tool for Intrapartum Care in Rural Kenya: Observational Analysis

**DOI:** 10.2196/34741

**Published:** 2022-06-20

**Authors:** Nhi Dinh, Smisha Agarwal, Lisa Avery, Priya Ponnappan, Judith Chelangat, Paul Amendola, Alain Labrique, Linda Bartlett

**Affiliations:** 1 Department of International Health Johns Hopkins Bloomberg School of Public Health Johns Hopkins University Baltimore, MD United States; 2 Centre for Global Public Health University of Manitoba Winnipeg, MB Canada; 3 Vecna Cares Burlington, MA United States; 4 Vecna Cares Kilgoris Kenya

**Keywords:** newborn, neonatal health, maternal health, intrapartum care, labor and delivery, Kenya, digital clinical decision support, health information systems, digital health, implementation research

## Abstract

**Background:**

iDeliver, a digital clinical support system for maternal and neonatal care, was developed to support quality of care improvements in Kenya.

**Objective:**

Taking an implementation research approach, we evaluated the adoption and fidelity of iDeliver over time and assessed the feasibility of its use to provide routine Ministry of Health (MOH) reports.

**Methods:**

We analyzed routinely collected data from iDeliver, which was implemented at the Transmara West Sub-County Hospital from December 2018 to September 2020. To evaluate its adoption, we assessed the proportion of actual facility deliveries that was recorded in iDeliver over time. We evaluated the fidelity of iDeliver use by studying the completeness of data entry by care providers during each stage of the labor and delivery workflow and whether the use reflected iDeliver’s envisioned function. We also examined the data completeness of the maternal and neonatal indicators prioritized by the Kenya MOH.

**Results:**

A total of 1164 deliveries were registered in iDeliver, capturing 45.31% (1164/2569) of the facility’s deliveries over 22 months. This uptake of registration improved significantly over time by 6.7% (SE 2.1) on average in each quarter-year (*P=*.005), from 9.6% (15/157) in the fourth quarter of 2018 to 64% (235/367) in the third quarter of 2020. Across iDeliver’s workflow, the overall completion rate of all variables improved significantly by 2.9% (SE 0.4) on average in each quarter-year (*P*<.001), from 22.25% (257/1155) in the fourth quarter of 2018 to 49.21% (8905/18,095) in the third quarter of 2020. Data completion was highest for the discharge-labor summary stage (16,796/23,280, 72.15%) and lowest for the labor signs stage (848/5820, 14.57%). The completion rate of the key MOH indicators also improved significantly by 4.6% (SE 0.5) on average in each quarter-year (*P*<.001), from 27.1% (69/255) in the fourth quarter of 2018 to 83.75% (3346/3995) in the third quarter of 2020.

**Conclusions:**

iDeliver’s adoption and data completeness improved significantly over time. The assessment of iDeliver’ use fidelity suggested that some features were more easily used because providers had time to enter data; however, there was low use during active childbirth, which is when providers are necessarily engaged with the woman and newborn. These insights on the adoption and fidelity of iDeliver use prompted the team to adapt the application to reflect the users’ culture of use and further improve the implementation of iDeliver.

## Introduction

### Background

Kenya has made major strides in preventing maternal deaths, reducing the maternal mortality ratio by half (52%) from 708 maternal deaths per 100,000 livebirths in 2000 to 342 maternal deaths per 100,000 livebirths in 2017 [1]. To promote the use of maternity services and reduce pregnancy-related mortality, a maternal health care policy was implemented in 2013 to abolish fees associated with childbirth in all public health facilities, which resulted in an increase in facility-based deliveries by 29.5%, from 234,601 deliveries before policy implementation to 303,705 deliveries after policy implementation [2]. However, this improved coverage of hospital-based births alone did not yield as much gain in maternal and newborn survival as expected, spurring additional efforts to improve quality of care and achieve Sustainable Development Goal 3.1—to reduce maternal mortality to ≤140 per 100,000 live births by 2030 [2-4].

The first Kenya Ministry of Health (MOH) report on Confidential Enquiry into Maternal Deaths noted that constraints to quality care that were contributing factors to most maternal deaths included delays in initiation of treatment, inadequate clinical skills, insufficient quality monitoring, and poor record keeping and documentation [5,6]. These issues are heavily affected by the underlying factors of understaffing with its associated burnout and fatigue and lack of adequate resources including space, privacy, training, and commodities [7]. Addressing these challenges could improve maternal and neonatal outcomes in Kenya and countries with similar challenges and resource constraints. Bhutta et al [8] reported that among all women giving birth in health facilities worldwide, if 90% of them actually received the recommended interventions during labor and delivery, an estimated 84% (113,000) of maternal deaths, 76% (531,000) of stillbirths, and 77% (1.325 million) of neonatal deaths can be prevented globally. This is supported by other studies reporting that interventions delivered during labor and childbirth provided the maximum benefits to avert stillbirths and neonatal and maternal deaths [9-11]. Similar projections based on Kenya’s data from 1990 to 2015 showed that active management of the third stage of labor and treatment of eclampsia accounted for 86% of maternal lives saved, and optimal care during childbirth and the postnatal period accounted for 70% of neonatal deaths averted [12].

Digital health interventions have the potential to improve adherence to recommended protocols by care providers during labor and delivery and to positively impact maternal and neonatal health outcomes [13]. A study on skilled birth attendants in Kenya showed that users of electronic partographs, in comparison with users of paper partographs, were more likely to be compliant with a set of standard practices during labor and delivery, including measuring pulse, temperature, amniotic fluid status, molding, blood pressure, and urine [14]. The use of electronic partographs was also associated with 56% reduction in suboptimal newborn outcomes [14]. According to the World Health Organization’s (WHO) recommendations on digital interventions for health system strengthening, the digital monitoring of clients’ health status and health service use has the potential to improve continuity and timely provision of care and adherence to clinical guidelines [15].

### Objective

Within this context, formative research was undertaken to assess the need and design of a potential digital solution, resulting in the development of iDeliver—a clinical decision support intervention developed by Vecna Cares, Johns Hopkins Bloomberg School of Public Health, and Scope (formerly known as M4ID) and supported by Merck for Mothers. Guided by human-centered design priorities elicited from stakeholders, the intended use of iDeliver was to help health care workers navigate the assessment and triage of pregnant clients on arrival at a facility, guide clinical decision-making during labor and delivery and immediately after childbirth, and streamline data reporting processes. If used correctly and efficiently, iDeliver has the potential to strengthen the quality of pregnancy, childbirth, and newborn care and facilitate the improvement of maternal and newborn health outcomes. This study takes an implementation research approach to evaluate the adoption and fidelity of iDeliver.

Using the iDeliver digital platform implemented at the Transmara West Sub-County Hospital in Kenya, we leveraged routinely collected data in a novel way to assess, quantify, and drive improvements in the *actual use *of this digital intervention by examining the completeness of the data collected. Although noted as a highly useful step in the Monitoring and Evaluating Digital Interventions guide [16], mining routinely collected data is a neglected area of digital implementation—a critical *middle ground* between the frequently assessed *acceptability* and *impact* of digital systems [16]. Acceptability is a necessity, but needs to be translated to use, which is the mediator of impact.

For the purpose of this study, adoption was defined as the uptake of iDeliver [17], and fidelity was defined as the degree to which iDeliver was used as it was designed originally [17], following the definition of implementation outcome variables described by Peters et al [17]. We also examined the data completeness of priority indicators to monitor maternal and newborn health outcomes and quality of care identified by the Kenya MOH’s Reproductive and Maternal Health Services [18-21] to assess the feasibility of using iDeliver to provide routine reports to the MOH. This study provided insights on variability in iDeliver use over time and identified areas for improvements and successful deployment strategies. Through this paper, we present a proof of concept of using a decision support tool to support documentation and clinical decision-making around prenatal, intrapartum, and postnatal care. In the real-world setting with limited resources, this process of evaluating implementation outcomes is a crucial intermediate step for a novel digital health intervention such as iDeliver to achieve its ultimate goal of improving maternal and newborn health outcomes. The methods used and corresponding findings could be helpful to other digital health solution implementors during similar early stages of technology deployment.

## Methods

### iDeliver—Description of the Technology

**Figure 1:**
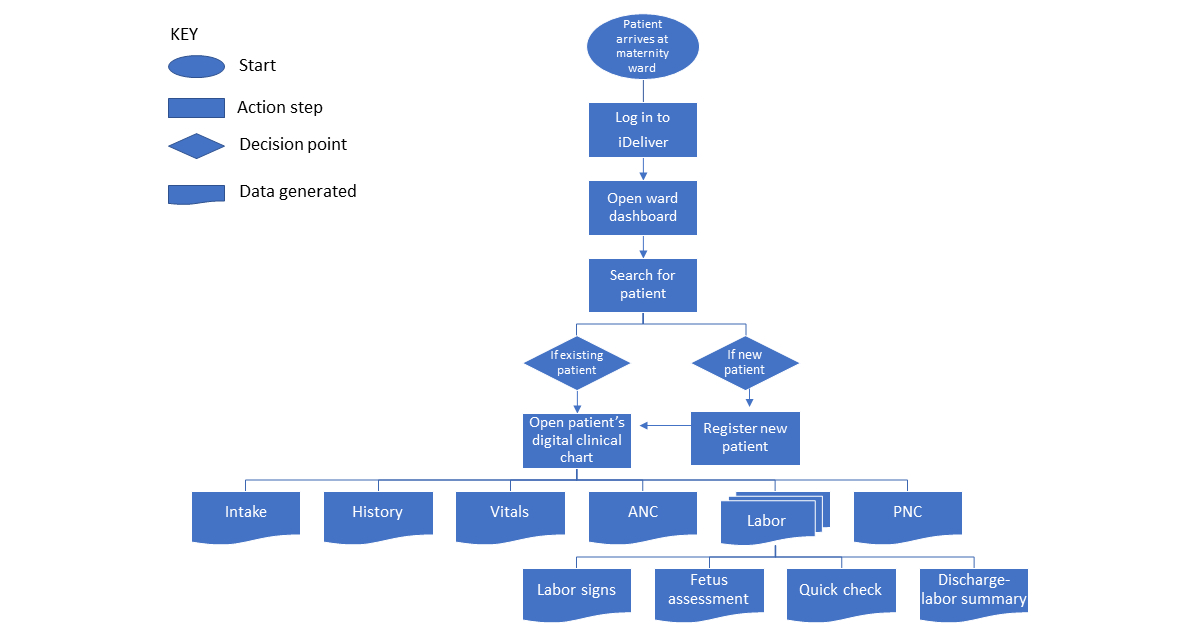
User’s workflow through iDeliver when a patient arrives at the maternity ward for labor and delivery. ANC: antenatal care; PNC: postnatal care.

iDeliver is a software application that allows health care providers to document relevant patient information and clinical progression throughout the continuum of maternal care in real time. Figure 1 summarizes the overall workflow. The version of iDeliver assessed in this study focused on intrapartum care; recent updates to the application also include antenatal and postnatal care components. The health care provider registers a new patient when she arrives at the labor and delivery ward and enters the key patient demographic and clinical information, which generates an acuity score for triage priority. All active registered patients can be seen on a dashboard from which users can access a patient’s digital clinical chart, navigate to any section—intake, history, vital signs, labor signs, fetal assessment, and discharge-labor summary—and enter the patient’s information at successive appointments to maintain a longitudinal health record. Digital clinical decision support algorithms and patient management guidelines for iDeliver are based on WHO’s Managing Complications in Pregnancy and Childbirth [22], Better Outcomes in Labour Difficulty Initiative [23], and Recommendations for Intrapartum Care for a Positive Childbirth Experience [24]. In addition, iDeliver includes clinical training resources, electronic medical record function, and report generation. Further information on the design, development, and implementation of iDeliver has been presented elsewhere [25].

### Site Selection

iDeliver was developed in collaboration with nurses, midwives, physicians, and public health administrators in Transmara West and Transmara East Sub-Counties of Narok County, Kenya. It was first implemented in 2017 at the Transmara West Sub-County Hospital in Kilgoris, which is a level-4 tertiary facility offering comprehensive emergency obstetric and newborn care services, with an average of 1150 births annually. It has since been scaled up to 13 other sites in Kenya and Tanzania. This study focused on iDeliver implementation at the Transmara West Sub-County Hospital during the 22-month period after transition to OpenMRS (OpenMRS Inc) platform (December 2018 to September 2020).

### Technology Introduction and Training

Since deployment, the application underwent significant updates. Transition from a proprietary to an open-source back end—OpenMRS—was done in November 2018. iDeliver interfaces were built as modular, encapsulated setup code built upon the OpenMRS application platform using ReactJS (Meta), a modern front-end language.

As of September 2020, 5 physicians and 14 nursing officers at the Transmara West Sub-County Hospital were trained to use iDeliver, with 40% (2/5) of the physicians and 57% (8/14) of the nursing officers as current active users. User training was conducted on site every 6 months to account for any upgrades in the application and for staff rotation. Training of new staff occurred on an as-needed basis.

### Analytic Approach

Data were extracted and deidentified using MySQL (version 5.6.49; Oracle Corporation). Then, MySQL Workbench (version 8.0; Oracle Corporation) was used to export the data into Excel format. All statistical analyses were performed using R (version 4.0.2; R Foundation for Statistical Computing).

We conducted descriptive analysis to summarize the characteristics of all mothers and newborn infants with information registered in iDeliver within the study period. Then, we assessed iDeliver’s adoption by exploring the following: (1) what proportion of services provided at the health facility are captured by iDeliver and (2) does the uptake of iDeliver use improve over time? To answer the first question and measure iDeliver’s uptake, we divided the number of deliveries registered in iDeliver by the number of deliveries recorded on paper at the Transmara West Sub-County Hospital from December 2018 to September 2020. To answer the second question, we assessed the trends in the uptake of iDeliver, by quarter-year, using simple linear regression. A *P* value of <.05 was considered as statistically significant.

We assessed the fidelity of iDeliver use to its original purpose as a decision-making and data management tool by examining which feature or features of iDeliver are used most by users, as assessed by data completion. We used the proportion of data available across the labor and delivery workflow to identify both areas of high use and missed opportunities for use. In particular, we assessed data completion for each stage of the iDeliver’s labor and delivery workflow: (1) intake, (2) history, (3) vital signs, (4) labor signs, (5) fetus assessment, (6) quick check, and (7) discharge-labor summary to identify the aspects of the intrapartum process that were plausible for care providers to use and if the use reflected iDeliver’s envisioned function for intrapartum clinical guidance. We also assessed the data completion for each stage over time, by quarter-year, using simple linear regression. A *P* value of <.05 was considered as statistically significant.

To assess the feasibility of using iDeliver to provide routine reports based on the priority indicators to monitor maternal and newborn health outcomes and quality of care identified by the Kenya MOH’s Reproductive and Maternal Health Services [18-21], we also examined the data completeness of those indicators from the MOH’s maternal and perinatal notification and review forms that overlapped with the data in iDeliver. These indicators were referral information (referral from community unit or health facility or referral out to community unit); mother’s HIV status; parity; fetal presentation; mode of delivery; date and time of delivery; sex of baby; condition of baby at birth; appearance, pulse, grimace, activity, and respiration score (at 1, 5, and 10 minutes); baby given tetracycline; condition of mother; and condition of baby at discharge. We also assessed the data completeness of these indicators over time, by quarter-year, using simple linear regression. A *P* value of <.05 was considered as statistically significant.

### Ethics Approval

The study was approved by the institutional review board of Johns Hopkins Bloomberg School of Public Health (protocol code I18203; December 8, 2021).

## Results

### Overview

Data from a total of 1164 deliveries were included in this analysis, spanning 22 months from December 2018 to September 2020. On average, the registered mothers were aged 24.1 years, with a median parity of 1 (range 0-9; Table S1 in Multimedia Appendix 1). Most mothers (996/1164, 85.57%) did not have their education information recorded in iDeliver. Of the 1164 mothers with information recorded, 394 (33.85%) had at least four antenatal visits before their delivery. Table S2 in Multimedia Appendix 2 summarizes the outcomes of the deliveries registered in iDeliver during the study period. Of the 1164 deliveries recorded, most infants were born at term (n=827, 71.05%), with a normal birth weight (n=843, 72.42%), by spontaneous vaginal delivery (n=879, 75.52%). In total, 3.44% (40/1164) of the births were classified as stillbirths (19/40, 48% fresh and 21/40, 52% macerated). Of the 976 babies who were born alive, 943 (96.6%) babies had their vital signs status recorded on discharge—941 (96.4%) were classified as alive and 2 (0.2%) were classified as dead. There was no information on condition at discharge for 3.4% (33/976) babies who were born alive. There was no record of mothers’ deaths (0/1164, 0% mothers were classified as dead, and 142/1164, 12.19% of the registration did not have this information recorded).

### Assessment of Implementation Outcome: Adoption of iDeliver

**Figure 2:**
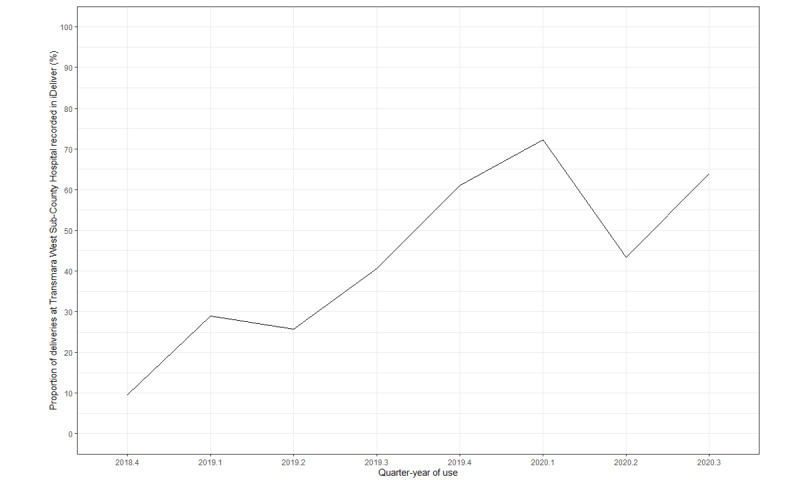
Proportion of deliveries captured in iDeliver compared with paper-based records in the Transmara West Sub-County Hospital, Kenya, by quarter-year from December 2018 to September 2020.

Over the 22 months, on average, 45.31% (1164/2569) of the deliveries captured in the existing paper-based record were recorded in iDeliver. The uptake increased by 6.7% on average in each quarter-year, from 9.6% (15/157) in the fourth quarter of 2018 to 64% (235/367) in the third quarter of 2020 (*β*_1_=6.7; SE 2.1; *P*=.005; *R*^2^=0.3). Figure 2 shows the overall increasing trend in the proportion of deliveries recorded by the Transmara West Sub-County Hospital between December 2018 and September 2020, by quarter-year. In the second quarter of 2020, we saw a sharp decline in adoption—a change attributed by local staff to the effect of the COVID-19 epidemic on hospital births. Then, adoption increased again in the third quarter of 2020.

### Assessment of Implementation Outcome: Fidelity of iDeliver Use Across the Labor and Delivery Workflow

**Figure 3:**
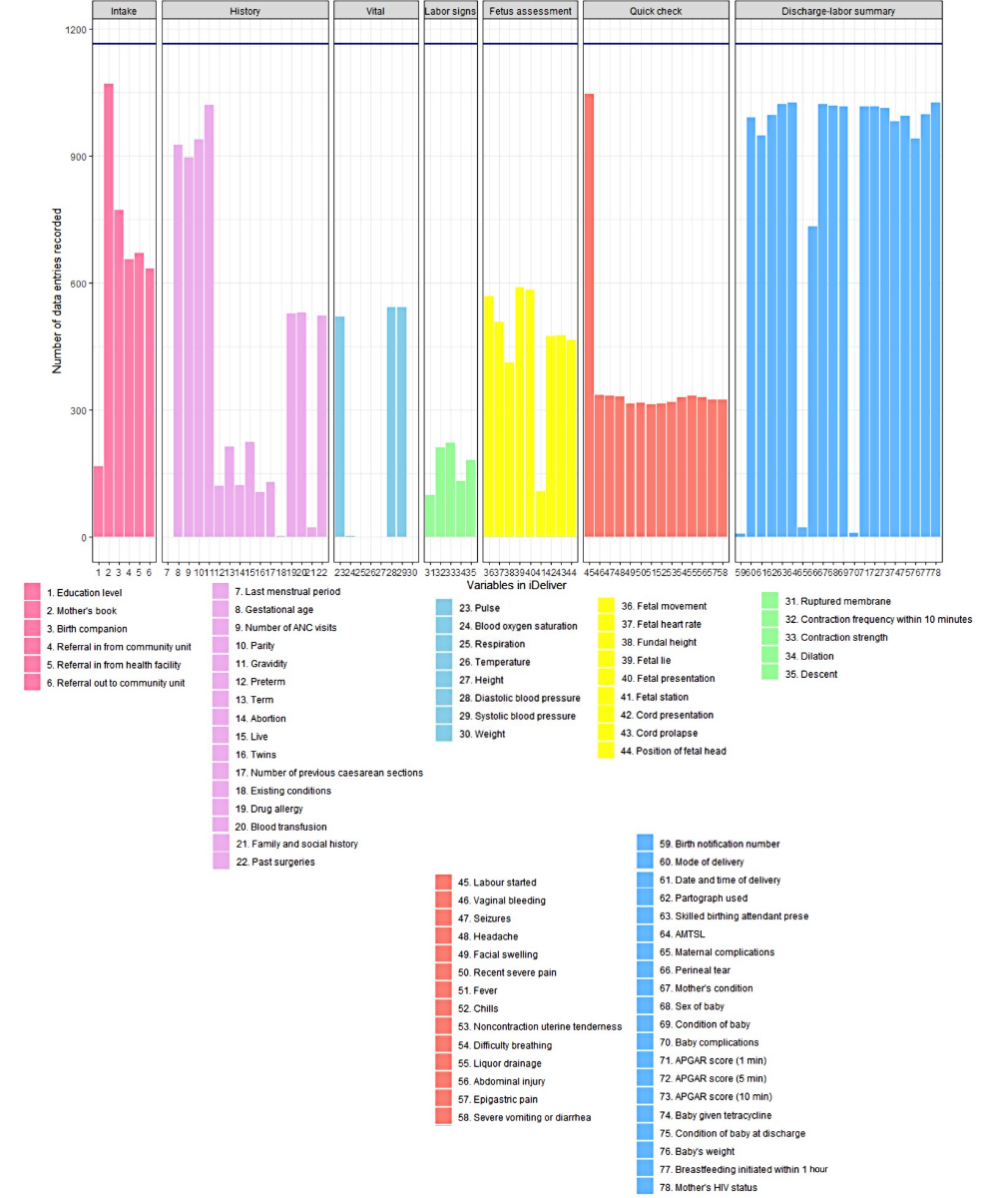
Summary of data completion for all variables at each stage of the iDeliver workflow among all deliveries registered in iDeliver at the Transmara West Sub-County Hospital from December 2018 to September 2020 (N=1164). AMTSL: active management of the third stage of labor; ANC: antenatal care; APGAR: appearance, pulse, grimace, activity, and respiration.

Figure 3 summarizes the use of iDeliver by each stage of the labor and delivery workflow over the 22 months of data, combined: (1) intake, (2) history, (3) vital signs, (4) labor signs, (5) fetus assessment, (6) quick check, and (7) discharge-labor summary. Of the 1164 deliveries registered in iDeliver from December 2018 to September 2020, the number of data entries was captured in each bar for variables that were organized into these 7 stages. The discharge-labor summary stage of iDeliver had the best data completion rates for all variables within this stage at 72.15% (16,796/23,280), followed by the intake stage (3969/6984, 56.83%), fetus assessment stage (4187/10,476, 39.97%), history stage (6301/18,624, 33.83%), and quick check stage (4945/15,132, 32.68%; Table 1). The data completion rates were lowest for vital signs (1607/9312, 17.26%) and labor signs stages (848/5820, 14.57%; Table 1).

Overall, the completion rate of all variables improved significantly, by 2.9% on average in each quarter-year, from 22.25% (257/1155) in the fourth quarter of 2018 to 49.21% (8905/18,095) in the third quarter of 2020 (*β*_1_=2.9; SE 0.4; *P*<.001; *R*^2^=0.03). Table 1 also summarizes results from the linear regression analysis assessing the change in data completion of the iDeliver workflow stages over time. Significant increases in data completion in each quarter-year were observed in the history, fetus assessments, quick check, and discharge-labor summary stages (Table 1).

**Table 1:** Change in the average percentage of data recorded for each stage of the iDeliver workflow at the Transmara West Sub-County Hospital, Kenya, by quarter-year from December 2018 to September 2020.

Labor and delivery stage of the iDeliver workflow	Data recorded from December 2018 to September 2020, %	Change in percentage of data recorded by quarter-year, *β*_1_ (SE)	*P* value
Intake	56.8	−1.2 (1.3)	.40
History	33.8	3.1 (0.9)	<.001
Vital signs	17.3	1.2 (1)	.20
Labor signs	14.6	−1.4 (0.7)	.07
Fetus assessment	40	3.6 (0.8)	<.001
Quick check	32.7	2.6 (0.6)	<.001
Discharge-labor summary	72.1	5.7 (0.7)	<.001
All variables in iDeliver	41.1	2.9 (0.4)	<.001

### Assessing the Quality of Indicators From the Kenya MOH’s Reproductive and Maternal Health Services

**Figure 4:**
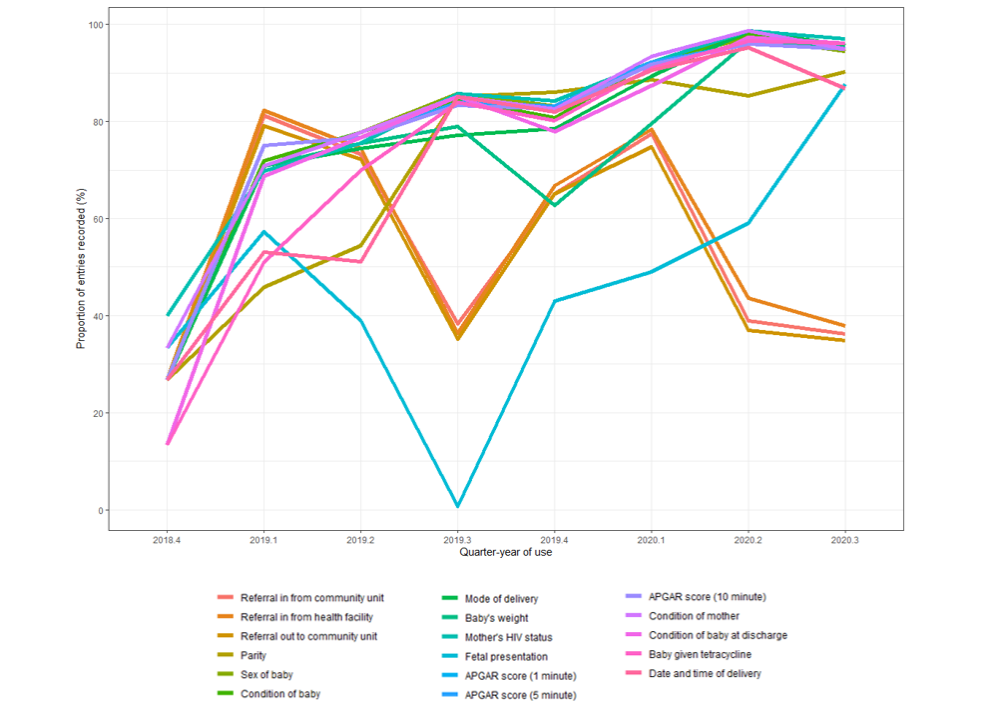
Proportion of data recorded for indicators that are prioritized by the Kenya Ministry of Health in iDeliver at the Transmara West Sub-County Hospital, Kenya, from November 2018 to September 2020. APGAR: appearance, pulse, grimace, activity, and respiration.

The average data completion rate of the Kenya MOH’s key obstetric care indicators increased significantly by 4.6% on average in each quarter-year (*β*_1_=4.6; SE 0.5; *P*<.001; *R*^2^=0.2), from 27.1% (69/255) in the fourth quarter of 2018 to 83.75% (3346/3995) in the third quarter of 2020. The indicators that showed the greatest improvements in completion rate over time were sex of baby; condition of baby at birth; mode of delivery; baby’s weight; appearance, pulse, grimace, activity, and respiration scores (at 1, 5, and 10 minutes); baby given tetracycline; condition of baby at discharge; and date and time of delivery—all of these indicators reached completion rate >80% by the third quarter of 2020 (Figure 4). The data completion rate for the fetal presentation indicator showed a steep decrease from the first to the third quarter of 2019 and steadily improved again thereafter (Figure 4).

## Discussion

### Principal Findings

#### Overview

The data captured by iDeliver indicate that its fidelity and adoption by health workers at the Transmara West Sub-County Hospital tertiary health care facility in rural Kenya showed substantial improvement: use of iDeliver at the Transmara West Sub-County Hospital increased over the 22 months of implementation, by 6.7% on average (SE 2.1) in each quarter-year, from 9.6% (15/157) in the fourth quarter of 2018 to 64% (235/367) in the third quarter of 2020. Data quality also improved over time as the average data completion rate across all variables increased significantly in each quarter-year and for 4 of the 7 stages of iDeliver’s workflow. The additional analysis on the data quality of a subset of variables that overlapped with the MOH’s maternal and perinatal death notification and review forms also reflected this longitudinal trend of improvement in most variables.

This upward trend in adoption can be attributed to the initial human-centered design efforts, numerous trainings with on-site training on iDeliver updates every 6 months, accounting for staff rotation, full-time on-going support by field staff based in Transmara, and the adaptations made to the iDeliver platform to be responsive to user requests. The uptake of iDeliver, as measured by the proportion of deliveries at the facility that was recorded in the application, decreased briefly in the second quarter of 2020 (Figure 2), reported as the impact of the COVID-19 pandemic, which caused disruption of routine health services including maternal care in Kenya and worldwide [26]. In addition, health care worker strikes occurred in Kenya during this challenging time [27].

A potential reason for the highest data completion rate at the discharge-labor summary stage of iDeliver is that the monthly reports to the District Health Information Software and information for facility billing can be easily generated from iDeliver’s discharge-labor summary page. The observed pattern of less iDeliver use during active labor is further explained through an internal evaluation of iDeliver that assessed its usability, acceptability, functionality, effectiveness, and sustainability [28]. Health provider interviews noted that most users entered the data after delivery because they could not input data during a delivery owing to short staffing and that the mother and baby required their full attention until the delivery was complete or *until the gloves come off* [28]. Although this is understandable given the context, it also indicates that the opportunity to use iDeliver for real-time clinical decision-making during delivery is challenging. Patterns of data completeness suggested that users might have preferred iDeliver’s feature as a medical record keeping tool and to use it after the active labor and delivery time. On the basis of these findings, the iDeliver team adapted the application to reflect the providers’ use culture: a complication management checklist was implemented, modeled after the WHO Surgical Safety Checklist [29], to facilitate a rapid review of the key care principles before or during complication management and the postmanagement documentation. Exploring alternative methods to input data during active labor such as a stylus that can be sterilized, a stylus or tablet inserted into a sterile plastic shield, or dictation capacity can also assist providers with ease of use. Additional sources for clinical guidance have been added to iDeliver so that providers can review global standards of care principles before or after managing a patient with complications, including a web-based version of WHO’s Managing Complications in Pregnancy and Childbirth [22], the Merck Manual video library, and the Safe Delivery App [30].

Although the data quality improved over time, completion rates for the vital signs, fetus assessment, labor signs, and quick check stages were still <50%. Checking the vital signs (maternal pulse, blood pressure, and temperature) is important to assess women’s well-being during labor and can indicate early signs of complications such as pre-eclampsia, early intrapartum hemorrhage, or impending infection. Examining the progress of labor is necessary to determine whether a woman has inadequate, prolonged, or obstructed labor—all of which can contribute to maternal morbidity and mortality and fetal death. The data missing in the digital system were mostly captured in paper-based records. However, if iDeliver was the only system that captured the data and more than half of the mothers did not have this critical clinical information reported digitally, maternal and fetal welfare could be compromised. Maintaining and improving timely and complete data records in a system such as iDeliver is crucial to capture concrete issues for decision makers to address suboptimal maternal and neonatal outcomes.

Data from iDeliver’s aforementioned internal evaluation showed that users valued the benefits of iDeliver for medical record keeping and data storage [28]—a preference also reflected in our analysis, as the labor and delivery summary was completed most by users. Reporting of these indicators also improved significantly over time. Most of this information overlapped with the indicators that we assessed based on the MOH’s maternal and perinatal death notification and review. Furthermore, the reported 43 (3.51%) stillbirths out of 1224 deliveries yields a stillbirth ratio of 35:1000 total births—a figure similar to a previous observational study reporting facility-based stillbirth ratio at 38.8:1000 at Kenyan hospitals providing comprehensive emergency obstetric care [31]. These findings provide evidence to support iDeliver’s functionality as a platform to provide routine reports (replacing hand-calculated reports) and other key information on quality of care to the facility administrator and the district and national health authority. Users also found that iDeliver’s function as an electronic medical record made data extraction for quality improvement audits and health management information systems easier [28].

A use assessment of an intrapartum digital clinical decision support system (CDSS) at the participating facilities in Tanzania and Ghana over 20 months showed that 83% and 67% of all deliveries, respectively, were recorded [32]. In comparison, over 22 months, iDeliver recorded data from 45.31% (1164/2569) of all deliveries overall, with improved uptake from 9.6% (15/157) in the fourth quarter of 2018 to 64% (235/367) in the third quarter of 2020—a figure close to the CDSS assessed in Ghana, in particular. Other studies assessing the implementation of digital CDSSs in Burkina Faso and India identified common challenges: increased burden for health care providers owing to lack of staff; dual documentation requirements; non–user-friendly platform; high staff turnover; and lack of integration with clinical workflow, continuous training, and staff’s motivation [32-34]. On the basis of these insights and the findings from this analysis, strategies to address the implementation challenges facing iDeliver include the following:

#### Phasing Out Paper-Based Records

The introduction of a digital tool may add to the heavy workload that care providers are carrying, as users need to input data into the new digital system in addition to the paper-based system. This parallel system of double entry was reported as the least-liked aspect of the iDeliver implementation by users, posing the greatest challenge to the application’s adoption [28]. To alleviate this burden of work for care providers and improve the adoption, working with facility administrators to phase out paper-based records is a promising strategy. Our experience in Zanzibar supports this premise: a paperless policy was enacted in the major maternity hospital in Zanzibar shortly after iDeliver was implemented there, and birth registrations in iDeliver increased to 100% quickly (Saye, J, unpublished data, April 2022). Furthermore, the significant improvement in data quality of indicators prioritized by the MOH indicates the potential of iDeliver’s functionality as a platform to provide key information to facility administrators and replace hand-calculated routine reports to the MOH. Identifying the key indicators, the completion of which must be focused on, such as the MOH indicators, will prioritize data entry and enhance iDeliver’s support to quality of care. In October 2020, this strategy was tested by making the MOH indicators mandatory in iDeliver at the Transmara West Sub-County Hospital. The only way for users to not complete these indicators is to skip the whole section that contains the mandatory MOH indicators. The preliminary analysis shows that the average data completeness rate of the MOH indicators increased to 92% after the update. In addition, the adoption of iDeliver also improved, with 88.9% (210/236) of the total deliveries at the Transmara West Sub-County Hospital being captured in iDeliver from October 2020 to April 2021. This improvement further supports the potential to phase out paper-based records to fully adopt iDeliver as the hospital’s main medical record system.

#### Enhancing the Medical Record Function

Insight from both routinely collected data from iDeliver and interviews from users [28] suggest the preference for using iDeliver as an electronic medical record tool. In October 2020, plans were made to implement antenatal and postnatal care modules in iDeliver at the Transmara West Sub-County Hospital, allowing users to keep records of the whole continuum of maternal care for every patient. A dedicated record keeping staff might be considered, but likely difficult to scale-up, given the already widespread human resource challenges.

#### Enhancing the Clinical Decision Support Function

Even though iDeliver was initially intended as an intrapartum tool, the low coverage of data entry in the vital signs, labor signs, fetal assessments, and quick check stages suggests limited applicability as a clinical decision support tool for intrapartum care in the current context. Additional implementation research is in progress to understand the parameters that health care providers use for clinical decision-making and to derive user-based solutions to adapt iDeliver and improve its usability.

#### Increasing Motivation for Users

Although the system currently includes user credentials, users at the hospital share devices and often do not log out of their accounts. With this culture of use, we could not assess which type of provider—nurses, midwives, or physicians—used iDeliver the most. Future training of iDeliver should encourage users to log in to the system with their unique credentials so that strengths and areas for improvement for each user can be addressed, which could enhance individual user’s accountability. This practice could help identify users who are iDeliver champions—users who use the application diligently and who could train or motivate others. An evaluation of a digital device to reduce maternal mortality and morbidity in low-resource settings reported that the identification and training of key champions, who were clinical staff members who received in-depth training and could support others, was the key implementation strategy enabling the feasibility of that novel intervention [35]. Other evaluation reports from India, Lao, Kenya, and Nigeria also found that staff motivation, satisfaction, confidence, and financial incentives are key factors to enable and sustain the use of novel digital health interventions [34,36,37].

### Limitations of This Study

Our study has the strength of leveraging routinely collected data from a newly developed and implemented application over a 22-month period to understand the potential use of an intrapartum decision support tool. However, as this was a retrospective study, these analyses were limited to only what the routinely collected data entailed. This limitation prevented us from conducting a systematic assessment of the behavioral, organizational, and technical determinants [38] of iDeliver’s implementation. Additional information from the literature, the field team’s insight, and the internal evaluation report were needed to provide insight on how data extracted directly from iDeliver reflected the intervention’s adoption and fidelity. In addition, owing to the challenge of data quality, we were not able to adequately assess the process outcomes related to quality of care and any link to maternal and neonatal outcomes. However, data missingness improved substantially over the first 22 months of implementation, and if this improvement persists, we anticipate using these data in the future to assess the impact of iDeliver on the quality of care and on maternal and neonatal outcomes.

### Conclusions

The results from this analysis provided us with an understanding of how iDeliver was implemented and used at the facility where it was first introduced. After 22 months, the adoption of iDeliver at the Transmara West Sub-County Hospital showed promising progress, as the use of the application increased and the data quality improved over time. These analyses also suggested that its function as a data collection and reporting tool was used more than its function as a clinical support tool, triggering the team to promptly adapt the application accordingly to reflect the users’ culture of use. This transition has the potential to further improve iDeliver’s use, enabling it to timely, correctly, and reliably capture data for both clinical and administrative decision-making support. The iDeliver team will continue to engage with the hospital administrators to support the transition to a paperless workflow and avoid the duplication of workload.

## Appendix

Multimedia Appendix 1 Characteristics of mothers with their deliveries registered in iDeliver at the Transmara West Sub-County Hospital, Kenya, from December 2018 to September 2020.

Multimedia Appendix 2 Summary of maternal and neonatal outcomes of deliveries registered in iDeliver at the Transmara West Sub-County Hospital, Kenya, from December 2018 to September 2020.

## Abbreviations

CDSS: clinical decision support system
MOH: Ministry of Health
WHO: World Health Organization

## References

[1] (2019). Maternal mortality: levels and trends 2000 to 2017. World Health Organization.

[2] Gitobu CM, Gichangi PB, Mwanda WO (2018). The effect of Kenya's free maternal health care policy on the utilization of health facility delivery services and maternal and neonatal mortality in public health facilities. BMC Pregnancy Childbirth.

[3] Chuma J, Maina T (2013). Free Maternal Care and Removal of User Fees at Primary-Level Facilities in Kenya: Monitoring the Implementation and Impact: Baseline Report. Health Policy Project.

[4] (2016). Standards for improving quality of maternal and newborn care in health facilities. World Health Organization.

[5] (2017). Saving Mothers’ Lives: Confidential Inquiry into Maternal Deaths in Kenya: First Report 2017. Ministry of Health, Republic of Kenya.

[6] Itote EW, Fleming LC, Mallinson RK, Gaffney KF, Jacobsen KH (2019). Knowledge of intrapartum care among obstetric care providers in rural Kenya. Int Health.

[7] Lusambili A, Wisofschi S, Shumba C, Obure J, Mulama K, Nyaga L, Wade TJ, Temmerman M (2020). Health care workers' perspectives of the influences of disrespectful maternity care in rural Kenya. Int J Environ Res Public Health.

[8] Bhutta ZA, Das JK, Bahl R, Lawn JE, Salam RA, Paul VK, Sankar MJ, Sankar JM, Blencowe H, Rizvi A, Chou VB, Walker N, Lancet Newborn Interventions Review Group, Lancet Every Newborn Study Group (2014). Can available interventions end preventable deaths in mothers, newborn babies, and stillbirths, and at what cost?. Lancet.

[9] Campbell OM, Graham WJ, Lancet Maternal Survival Series steering group (2006). Strategies for reducing maternal mortality: getting on with what works. Lancet.

[10] Ronsmans C, Graham WJ, Lancet Maternal Survival Series steering group (2006). Maternal mortality: who, when, where, and why. Lancet.

[11] Lee AC, Cousens S, Wall SN, Niermeyer S, Darmstadt GL, Carlo WA, Keenan WJ, Bhutta ZA, Gill C, Lawn JE (2011). Neonatal resuscitation and immediate newborn assessment and stimulation for the prevention of neonatal deaths: a systematic review, meta-analysis and Delphi estimation of mortality effect. BMC Public Health.

[12] Keats EC, Ngugi A, Macharia W, Akseer N, Khaemba EN, Bhatti Z, Rizvi A, Tole J, Bhutta ZA (2017). Progress and priorities for reproductive, maternal, newborn, and child health in Kenya: a countdown to 2015 country case study. Lancet Glob Health.

[13] Jo Y, Labrique AB, Lefevre AE, Mehl G, Pfaff T, Walker N, Friberg IK (2014). Using the lives saved tool (LiST) to model mHealth impact on neonatal survival in resource-limited settings. PLoS One.

[14] Sanghvi H, Mohan D, Litwin L, Bazant E, Gomez P, MacDowell T, Onsase L, Wabwile V, Waka C, Qureshi Z, Omanga E, Gichangi A, Muia R (2019). Effectiveness of an electronic partogram: a mixed-method, quasi-experimental study among skilled birth attendants in Kenya. Glob Health Sci Pract.

[15] (2019). WHO guideline: recommendations on digital interventions for health system strengthening. World Health Organization.

[16] (2016). Monitoring and evaluating digital health interventions: a practical guide to conducting research and assessment. World Health Organization.

[17] Peters DH, Adam T, Alonge O, Agyepong IA, Tran N (2013). Implementation research: what it is and how to do it. BMJ.

[18] (2017). Reproductive and Maternal Health Services: Maternal Death Notification Form (MDNF).

[19] (2016). Maternal Death Review Form.

[20] (2017). Reproductive and Maternal Health Services: Perinatal Death Notification Form (PDNF).

[21] (2017). Reproductive and Maternal Health Services: Perinatal Death Review Form (PDRF).

[22] (2017). Managing complications in pregnancy and childbirth: a guide for midwives and doctors. 2nd edittion. World Health Organization.

[23] Oladapo OT, Souza JP, Bohren MA, Tunçalp Ö, Vogel JP, Fawole B, Mugerwa K, Gülmezoglu AM (2015). WHO Better Outcomes in Labour Difficulty (BOLD) project: innovating to improve quality of care around the time of childbirth. Reprod Health.

[24] (2018). WHO recommendations: intrapartum care for a positive childbirth experience. World Health Organization.

[25] Bartlett L, Avery L, Ponnappan P, Chelangat J, Cheruiyot J, Matthews R, Rocheleau M, Tikkanen M, Allen M, Amendola P, Labrique A (2021). Insights into the design, development and implementation of a novel digital health tool for skilled birth attendants to support quality maternity care in Kenya. Fam Med Community Health.

[26] Busch-Hallen J, Walters D, Rowe S, Chowdhury A, Arabi M (2020). Impact of COVID-19 on maternal and child health. Lancet Glob Health.

[27] (2020). As Coronavirus Hits Africa, Doctors and Nurses Prepare to Strike. MEL Magazine.

[28] Avery L (2019). 2019 Internal Evaluation of iDeliver: Key Findings.

[29] WHO Surgical Safety Checklist. World Health Organization.

[30] The Safe Delivery App. Maternity Foundation.

[31] Aminu M, Bar-Zeev S, White S, Mathai M, van den Broek N (2019). Understanding cause of stillbirth: a prospective observational multi-country study from sub-Saharan Africa. BMC Pregnancy Childbirth.

[32] Sukums F, Mensah N, Mpembeni R, Massawe S, Duysburgh E, Williams A, Kaltschmidt J, Loukanova S, Haefeli WE, Blank A (2015). Promising adoption of an electronic clinical decision support system for antenatal and intrapartum care in rural primary healthcare facilities in sub-Saharan Africa: the QUALMAT experience. Int J Med Inform.

[33] Zakane SA, Gustafsson LL, Sie A, Tomson G, Loukanova S, Bastholm-Rahmner P (2017). Opportunities and obstacles using a clinical decision support system for maternal care in Burkina Faso. Online J Public Health Inform.

[34] Usmanova G, Gresh A, Cohen MA, Kim YM, Srivastava A, Joshi CS, Bhatt DC, Haws R, Wadhwa R, Sridhar P, Bahl N, Gaikwad P, Anderson J (2020). Acceptability and barriers to use of the ASMAN provider-facing electronic platform for peripartum care in public facilities in Madhya Pradesh and Rajasthan, India: a qualitative study using the technology acceptance model-3. Int J Environ Res Public Health.

[35] Vousden N, Lawley E, Nathan HL, Seed PT, Brown A, Muchengwa T, Charantimath U, Bellad M, Gidiri MF, Goudar S, Chappell LC, Sandall J, Shennan AH, CRADLE Trial Collaborative Group (2018). Evaluation of a novel vital sign device to reduce maternal mortality and morbidity in low-resource settings: a mixed method feasibility study for the CRADLE-3 trial. BMC Pregnancy Childbirth.

[36] Numair T, Harrell DT, Huy NT, Nishimoto F, Muthiani Y, Nzou SM, Lasaphonh A, Palama K, Pongvongsa T, Moji K, Hirayama K, Kaneko S (2021). Barriers to the digitization of health information: a qualitative and quantitative study in Kenya and Lao PDR using a cloud-based maternal and child registration system. Int J Environ Res Public Health.

[37] Akeju D, Okusanya B, Okunade K, Ajepe A, Allsop MJ, Ebenso B (2022). Sustainability of the effects and impacts of using digital technology to extend maternal health services to rural and hard-to-reach populations: experience from Southwest Nigeria. Front Glob Womens Health.

[38] Aqil A, Lippeveld T, Hozumi D (2009). PRISM framework: a paradigm shift for designing, strengthening and evaluating routine health information systems. Health Policy Plan.

[39] iDeliver - VecnaCares.

