# Supplementary material for: Implementation Outcomes Assessment of a Digital Clinical Support Tool for Intrapartum Care in Rural Kenya: Observational Analysis
Source: JMIR Form Res. 2022 Jun 20;6(6):e34741. doi: 10.2196/34741 (PMC9253974; doi:10.2196/34741)
Supplement: Multimedia Appendix 1 [file formative_v6i6e34741_app1.docx]

Multimedia Appendix 1

Table S1. Characteristics of mothers with their deliveries registered in iDeliver at the Transmara West Sub-County Hospital, Kenya, from December 2018 to September 2020.

|  | Total (n = 1164) | |
| --- | --- | --- |
|  | N | % |
| Maternal age at delivery, Mean (SD) | 24.1 (6.4) | |
| - Below 15 | 7 | 0.6 |
| - 15-19 | 198 | 17.0 |
| - 20-29 | 521 | 44.8 |
| - 30-39 | 154 | 13.2 |
| - 40 and above | 15 | 1.3 |
| - Not recorded | 269 | 23.1 |
| Mother’s Education |  |  |
| - Primary | 66 | 5.7 |
| - Secondary | 82 | 7.0 |
| - University | 8 | 0.7 |
| - Other Non-coded | 12 | 1.0 |
| - Not recorded | 996 | 85.6 |
| Parity |  | |
| - 0 | 301 | 25.9 |
| - 1 | 227 | 19.5 |
| - 2 | 170 | 14.6 |
| - 3 | 98 | 8.4 |
| - 4 | 56 | 4.8 |
| - 5+ | 87 | 7.5 |
| - Not recorded | 225 | 19.3 |
| Gravidity |  | |
| - 0 | 4 | 0.3 |
| - 1 | 378 | 32.5 |
| - 2 | 221 | 19.0 |
| - 3 | 174 | 14.9 |
| - 4 | 97 | 8.3 |
| - 5+ | 145 | 12.5 |
| - Not recorded | 145 | 12.5 |
| Number of ANC visits |  | |
| - 1 | 47 | 4.0 |
| - 2 | 124 | 10.7 |
| - 3 | 327 | 28.1 |
| - 4+ | 397 | 34.1 |
| - Not recorded | 269 | 23.1 |
