# Supplementary material for: Implementation Outcomes Assessment of a Digital Clinical Support Tool for Intrapartum Care in Rural Kenya: Observational Analysis
Source: JMIR Form Res. 2022 Jun 20;6(6):e34741. doi: 10.2196/34741 (PMC9253974; doi:10.2196/34741)
Supplement: Multimedia Appendix 2 [file formative_v6i6e34741_app2.docx]

Multimedia Appendix 2.

Table S2. Summary of maternal and neonatal outcomes of deliveries registered in iDeliver at the Transmara West Sub-County Hospital, Kenya, from December 2018 to September 2020.

|  | Total (n = 1164) | |
| --- | --- | --- |
|  | N | % |
| Sex of newborns |  | |
| - Female | 519 | 44.6 |
| - Male | 498 | 42.8 |
| - Not recorded | 147 | 12.6 |
| Gestational age at birth, Mean (SD) |  | |
| - Extremely Preterm (<28 weeks) | 6 | 0.5 |
| - Very preterm (28 – 31 weeks) | 8 | 0.7 |
| - Moderate to late preterm (32 – 36 weeks) | 84 | 7.2 |
| - Term (>= 37 weeks) | 827 | 71.0 |
| - Not recorded | 239 | 20.5 |
| Birth weight, Mean (SD) |  | |
| - Normal birth weight (>= 2500 g) | 843 | 72.4 |
| - Low birth weight (<2500) | 97 | 8.3 |
| - Not recorded | 224 | 19.2 |
| Mode of delivery |  | |
| - Spontaneous Vaginal Delivery | 879 | 75.5 |
| - Assisted Vaginal Delivery | 2 | 0.2 |
| - Breech | 8 | 0.7 |
| - Caesarian Section | 101 | 8.7 |
| - Not recorded | 174 | 14.9 |
| Mother’s condition at discharge |  | |
| - Alive | 1022 | 87.8 |
| - Dead | 0 | 0 |
| - Not Recorded | 142 | 12.2 |
| Baby’s condition at birth |  | |
| - Alive | 976 | 83.8 |
| - Fresh stillbirth | 19 | 1.6 |
| - Macerated stillbirth | 21 | 1.8 |
| - Not recorded | 148 | 12.7 |
| Baby’s condition at discharge | N = 976 | |
| - Alive | 941 | 96.4 |
| - Dead | 2 | 0.2 |
| - Not recorded | 33 | 3.4 |
